# The Diversity of Transplant Glomerulitis and its Relationship to Alloantibody

**DOI:** 10.1016/j.ekir.2026.106378

**Published:** 2026-02-25

**Authors:** Brian J. Nankivell, Seethalakshmi Viswanathan, Xinyi Ding, Jennifer S.Y. Li

**Affiliations:** 1 Department of Renal Medicine, Westmead Hospital, Sydney, New South Wales, Australia; 2 Department of Tissue Pathology and Diagnostic Oncology, Institute of Clinical Pathology and Medical Research, Sydney, New South Wales, Australia; 3 Westmead Institute for Medical Research, Sydney, New South Wales, Australia

**Keywords:** antibody-mediated rejection, donor-specific antibodies, glomerulitis, kidney transplantation

## Abstract

**Introduction:**

Although transplant glomerulitis often signifies antibody-mediated rejection (AMR), the pathophysiology of complement degradation split-product 4d (C4d) staining of peritubular capillaries (C4d_ptc_) and donor-specific antibodies (DSAs) negative cases is unclear.

**Methods:**

We compared Banff 1997 defined glomerulitis (*n* = 271, from 4300 biopsies) using serological and pathological dichotomization, against rejection without glomerulitis (g0REJ, *n* = 612) and normal controls (NIL, *n* = 1442).

**Results:**

Epidemiological risk factors were female recipients, living donation, early T-cell–mediated rejection (TCMR) or AMR, and previous corticosteroid rejection treatment. Glomerulitis associated with sensitization in 38.0%, ischemic endothelial injury in 20.7%, iatrogenic minimization in 30.3%, and nonadherence in 21.4%. Banff g score correlated with DSA+, C4d_ptc_+, endothelial C4d staining of glomerular capillaries positive (C4d_glom_+), Banff cg, i, t, ti scores, serum creatinine, and proteinuria. DSA+ glomerulitis was typified by inflammation, chronic scarring, C4d+, later presentation, treatment resistance, and functional deterioration (*P* < 0.001 vs. DSA−). DSA− glomerulitis from CD3+ TCMR (pure 43.3% and mixed 28.7% with AMR) presented earlier with excellent graft outcomes. C4d_ptc_− glomerulitis was etiologically classified TCMR in 36.8%, pure AMR in 28.1%, and mixed AMR in 31.3%. Isolated glomerulitis in 20.3% was diagnosed by surveillance sampling in 70.9%, displayed CD3+ in 51.9% and a distinct but minimal spatial transcriptomic phenotype, with superior allograft outcomes compared with glomerulitis with inflammation. Kidneys with glomerulitis and g0REJ failed more frequently than normal control samples (*P* < 0.001). Graft survival was independently determined by DSA+, C4d_ptc_+, transplant glomerulopathy, and chronic interstitial fibrosis using multivariable Cox regression, but not by glomerulitis score.

**Conclusion:**

Glomerulitis is a heterogenous biological lesion that frequently signifies AMR, however TCMR or innate cellular inflammation caused many DSA− and/or C4d_ptc_− episodes.

## Graphical abstract

**Figure:**
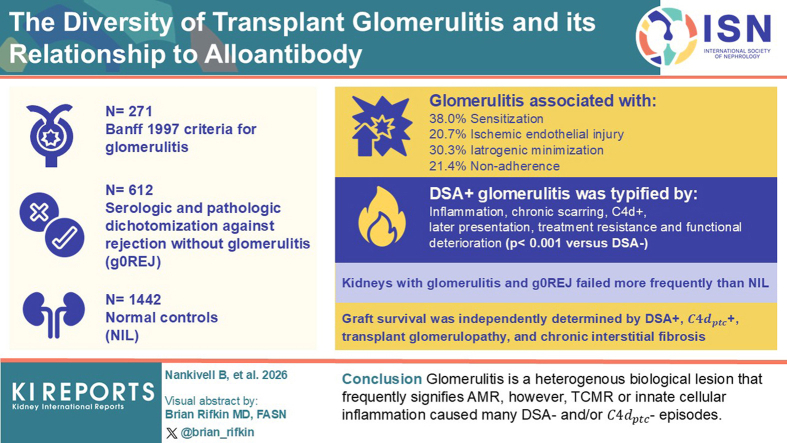

Transplant glomerulitis is a distinctive glomerulopathy characterized by intracapillary mononuclear cell accumulation, enlarged or necrotic endothelial cells, and occasional occluded glomerular capillaries^1^; and distinguished from native proliferative glomerulonephritis with extracapillary inflammation with glomerular tuft enlargement. Glomerulitis appears in hyperacute rejection from preformed antibody, early active AMR, and late chronic-active AMR with transplant glomerulopathy from DSA. Glomerulitis was incorporated into the Banff 1993 diagnostic AMR schema, with semiqualitative thresholds using percentage glomeruli affected (Banff g score) replacing imprecise “focal” and “diffuse” categories in 1997,^2^^,^^3^ and complete or partial capillary occlusion by leukocytes with endothelial cell enlargement (EOL) was added in 2013: a single leukocyte AND endothelial swelling causing ≥ 50% narrowing was stated to avoid overcalling circulating leukocytes as rejection.^4^^,^^5^ EOL correlates with glomerulitis score, serum creatinine, C4d_ptc_+, proteinuria, and subsequent glomerulopathy, but is rarely present in mild g1 (6.9%); making it unsuitable as a mandated exclusionary feature.^2^^,^^6^, ^7^, ^8^, ^9^ Glomerulitis and peritubular capillaritis constitute microvascular inflammation (MVI, defined as Banff g+ptc^3^ 2), which signify pathological cellular “activity.”^10^

There are many unknowns. Much of our fundamental knowledge comes from small historical studies which lacked precise pathological definitions, accurate solid-phase DSA, C4d, glomerulopathy, and outcome reporting. Some studies restricted analysis to Banff g2/3, causing selection bias.^11^^,^^12^ The cut-off number between normal and mild (endocapillary) “glomerulitis” was never validated in transplantation but appropriated from IgA glomerulonephritis at ≥ 5 (extracapillary) glomerular leukocytes.^2^^,^^8^ Interobserver reproducibility is low (κ; 0.2–0.3) with distinction between enlarged endothelial cells, adherent mononuclear cells, and adjacent extracapillary interstitial infiltrates challenging for reporting pathologists.^2^^,^^4^^,^^7^

The Banff AMR diagnostic triad uses glomerulitis to indicate acute organ injury (Banff g^3^ 1, criterion 1) and within conditional MVI^3^ 2 (Banff g+ptc^3^ 2) in the superseded Banff 2019 criterion 2 as a surrogate for antibody binding in C4d_ptc_− AMR.^13^^,^^14^ The specificity of glomerulitis for AMR diagnosis is challenged by its occurrence in recurrent and *de novo* glomerulonephritis, viral glomerulitis, and DSA− TCMR. Banff 2022 repositioned DSA−/C4d_ptc_− MVI^3^ 2 to diagnostically uncertain category,^10^ although many incomplete phenotypes cases are mild AMR recognized by unconventional subthreshold or shared-eplet DSA+, glomerular capillary loop C4d+, ultrastructural endothelial changes, and molecular classifiers, which demonstrate intermediate rates for subsequent active AMR, chronic glomerulopathy, and allograft loss.^10^^,^^15^, ^16^, ^17^, ^18^, ^19^, ^20^ The pathophysiology of DSA− and C4d_ptc_− glomerulitis and its interrelationships with AMR is similarly uncertain. Partial and incomplete phenotypes, including “isolated glomerulitis” are common but cannot be classified by the current Banff schema.

This study investigated the pathophysiology of glomerulitis to determine its relationship with AMR in a large, well-phenotyped biopsy cohort from ABO-compatible kidney transplant recipients. We evaluated the cause of DSA− and C4d_ptc_− glomerulitis as well as isolated glomerulitis using the Banff 2019 and 2022 AMR diagnostic criteria, and comprehensive etiological analysis using unconventional diagnostic classifiers.

## Methods

### Study Design and Clinical Groups

This single-center, observational study used prospective database with retrospective analysis of prespecified hypotheses. It was investigator-initiated and independent. Institutional ethics compliance included HREC LNR/12/WMEAD/114 (registered as “Understanding Transplant Injury”), 2019/STE12035, 2019/ETH09579 2019/ETH02085, 2025/ETH00055, and TPR2018/00040. A STROBE checklist is included (Supplementary Table S1). Consecutive, adequate specimens from May 2012 to May 2023, including indication biopsies, surveillance protocol (1-, 3-, and 12-month kidney plus 3-, 5-, 7-, and 10-year kidney-pancreas), and posttreatment verification samples were screened for inclusion. Unrelated conditions which activate complement (ABO-incompatible, atypical hemolytic uremic syndrome and thrombotic microangiopathy), provoke glomerular inflammation (recurrent or *de novo* glomerulonephritis), or alter chronic glomerular structure (e.g., diabetic nephropathy, amyloidosis) were excluded from the primary analysis. The study group included all Banff g ≥ 1 samples defined by Banff 1997 lesion criteria irrespective of DSA, C4d, and EOL status. Glomerulitis was compared with DSA− normal tissues (negative controls) and rejection cases without glomerulitis (g0REJ) as positive comparators.

### Pathological Assessment and Etiological Classification

Contemporaneous histology was reclassified to Banff 2022^13^ with Banff 2019 AMR for comparison using C4d_ptc_. Glomerulitis was defined using Banff 1997 to avoid selection bias against mild disease without EOL^3^ with glomerular inflammation defined by^3^ 5 glomerular leukocytes by 1 of 6 experienced departmental nephropathologists.^21^ Severity assessment used ordinal Banff g scores.^13^ C4d immunoperoxidase was used to score peritubular (C4d_ptc_); with additional nonconventional glomerular capillary loop staining (C4d_glom_) and endothelium and intima of muscular arteries (C4d_art_).^22^^,^^23^ C4d_glom_ used linear staining in ≥ 3 capillary loops: C4d_glom_1, faint and/or segmental in any glomerulus (≤9% glomeruli); C4d_glom_2, mild-to-strong intensity in 10% to 50% (segmental or global); C4d_glom_3, diffuse staining > 50% glomeruli. Nonspecific mesangial and arteriolar C4d staining was disregarded. C4d_glom_ background staining was absent in preimplantation donor tissue and minimal for C4d_ptc_ (2.1%) and C4d_art_ (3.5%).^22^ Banff AMR classification used C4d_ptc_^3^1 and DSA+ results.

Because many incomplete phenotypes of glomerulitis defy classification using the Banff schema, etiological classification used all available clinical, serological (including previous historical DSA and presensitization) and pathology using the “AMR triad” to assign causality.^24^ Exhaustive pathological review of glomerulitis included single and dual-stain CD3/CD68 immunophenotyping scored by a single blinded pathologist (SV), ultrastructural glomerular capillary endothelial abnormalities for endothelial cell hypertrophy and subendothelial widening (without neomembrane formation, “cg0e”),^16^^,^^17^ peritubular capillary multilayering ≥ 4 layers, and C4d_glom_^3^ 1 were also considered for classification.^25^^,^^26^

Exploratory glomerular spatial transcriptomics (VisiumHD, 10× Genomics, Pleasanton, CA) of isolated glomerulitis tissues (*n* = 5 glomeruli) was used to compare normal protocol biopsy negative controls (*n* = 7) and nephrectomy wedge samples of mixed AMR (*n* = 12, positive comparator), as described.^27^ Nonsclerosed glomeruli were manually selected by Loupe Browser (version 9.0, 10× Genomics, presented in detail in Supplementary Table S2), downstream analysis used R-studio with Seurat v5^28^ to identify differentially expressed genes at log2-fold > 0.5 and Benjamini-Hochberg adjusted *P* < 0.1,^28^ and gene set enrichment analysis of these differentially expressed genes by clusterProfiler.^29^ Isolated glomerulitis was defined by the absence of associated inflammation (Banff ti, i, ptc, v, and cg mandated zero).

For DSA testing on EDTA-treated sera, we used single Antigen Bead assays (LABScreen, One Lambda, Canoga Park, CA) incorporating human leukocyte antigen (HLA) class I (A, B, C) and class II (DRB1/3/4/5, DQ α/β, DPα/β) alleles characterized by 2-field sequence-based typing (Applied Biosystems, Thermo Fisher Scientific, CA) from 2017, replacing single-field molecular HLA typing by sequence-specific oligonucleotides. Laboratory defined DSA+ as median fluorescence intensity (MFI) ≥ 500).

### Statistical Analysis

Pearson’s or Spearman’s tests for correlations, principal component analysis for data reduction, and binomial generalized estimating equations for repeated samples were used. Multivariable models were constructed following backward elimination. For Kaplan-Meier and Cox regression, we used first glomerulitis index case from unique kidneys for time-to-event analysis. *P* values were 2-sided, a probability < 0.05 considered significant. Data are expressed as mean ± SD.

## Results

### Study Demographics and Epidemiology of Glomerulitis

**Figure 1:**
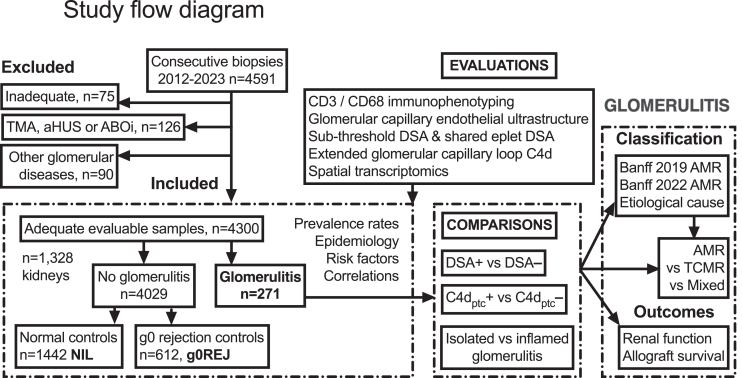
Study flow diagram. Numbers are biopsy samples and patients, except for graft outcome in glomerulitis which used first index biopsy in a unique kidney for actuarial survival. ABOi, ABO-incompatible; aHUS, atypical haemolytic uremic syndrome; AMR, antibody-mediated rejection; C4d, complement degradation split-product 4d; DSA, donor-specific antibody; g0REJ, all-cause rejection without glomerulitis (positive comparator); NIL, normal control samples; TCMR, T-cell–mediated rejection; TMA, thrombotic microangiopathy.

From 4591 consecutive kidney biopsies, exclusions included unsatisfactory tissue (*n* = 75), complement activating conditions, including atypical hemolytic uremic syndrome, ABO incompatible transplantation (*n* = 64, 7.8% with glomerulitis, C4d_glom_ 54.7%), or thrombotic microangiopathy (*n* = 126, 9.5% glomerulitis, C4d_glom_ 40.0%), proliferative glomerulonephritis (*n* = 74, 8.1% glomerulitis, C4d_glom_ 28.4%); and unrelated glomerular diseases (*n* = 16, 11.1% glomerulitis, C4d_glom_ 43.3%). The primary study group comprised 4300 samples with 3.2 ± 1.8 per kidney. Antibody subanalyses excluded cases without contemporaneous DSA (*n* = 694, mostly stable 3-month protocol) leaving 3806 (2474 with serology at biopsy and 1132 within 1 month) from 1250 recipients (Figure 1). Study patients were aged 47.5 ± 12.8 years, 62.9% male, 7.4% retransplanted, HLA Class I/II mismatched 3.9 ± 1.8 (of 6) and received a deceased donor kidney in 79.8% or kidney-pancreas in 30.3%. The allograft prevalence of glomerulitis was 15.4% (205/1328 kidneys), with 156 episodes occurring once and 49 on multiple occasions. Biopsy prevalence was 6.3% (271/4300) for indication in 52.8% (97.9% for dysfunction) or subclinical in 47.2% by protocol sampling. Kidneys with glomerulitis presented later with higher serum creatinine, urinary albumin-to-creatinine ratio, and MFI (*P* < 0.001 vs. no glomerulitis). The g0REJ (*n* = 612) group included Banff acute TCMR (plus borderline and grade II with arteritis, *n* = 367), active AMR (*n* = 29) chronic-active TCMR (*n* = 142) and chronic AMR (*n* = 74) as dominant diagnoses.

Cohort epidemiological analysis of clinical risk factors found that prevalent glomerulitis was predicted by living donation, early AMR and TCMR, and previous pulse corticosteroid rejection treatment using multivariable binomial generalized estimating equation (*n* = 4300 samples, 1327 patients).

### Etiological Classification of Glomerulitis

Rejection was the principal clinicopathological diagnosis in 66.8% of glomerulitis (181/271) cases compared with 15.2% without glomerulitis (612/4029, χ^2^ = 518.5, *P* < 0.001): which clinically presented as acute rejection (48.6%), subclinical rejection (18.8%), chronic AMR (20.4%), and CA-TCMR (12.1%). Other diagnoses were acute tubular injury (1.8%), BK virus allograft nephropathy (3.3%), interstitial fibrosis and tubular atrophy (11.8%), calcineurin nephrotoxicity (2.2%), and minor abnormalities (8.1%). Three glomerulitis cases could not be accurately classified. Seven early (0.58 ± 0.33 months) biopsies for delayed graft function (DGF) requiring dialysis showed mild and focal neutrophilic glomerulitis (all g = 1) with acute tubular injury which lacked DSA, Banff ptc, or C4d_ptc_. One showed segmental capillary thrombosis. Pulse corticosteroid treatment resolved glomerular inflammation verified by repeat sampling accompanying functional recovery over 2 to 8 weeks.

**Figure 2:**
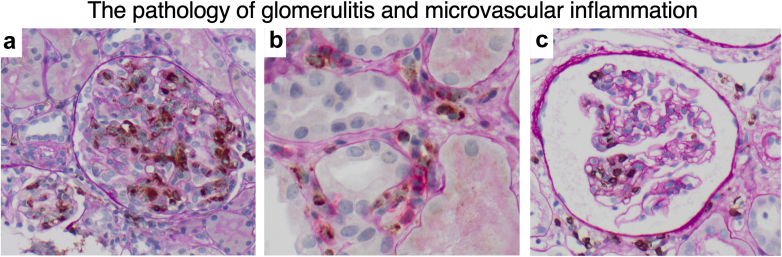
Infiltrating cells in glomerular microvascular inflammation. (a) DSA+ antibody-mediated rejection with glomerulitis. Brown CD68+ monocytes adhere to capillary endothelial cells with reactive enlargement and capillary loop luminal narrowing. CD3/CD68 dual-stain immunophenotyping (CD3+ cytoplasm is pink, magnification 200×). Periodic acid-Schiff counterstain for all images. (b) Peritubular capillaritis surrounding tubular cells. CD3/CD68 dual-stain (CD3+ pink, CD68+ brown, magnification 400×). (c) Segmental glomerulitis T cell (CD3+ brown for this image, magnification 200x) with endothelial cell swelling and luminal narrowing.

Of 261 remaining glomerulitis cases attributed to rejection, 26.1% (68/261) were classified as pure TCMR (including borderline); 49.2% using Banff 2022 TCMR criteria but increasing to 67.2% using comprehensive etiological classification of DSA−/C4d_ptc_− CD3+ glomerulitis with variable interstitial inflammation (Figure 2). AMR was diagnosed in 59.8% (156/261) using Banff 2019, 57.9% (151/261) using Banff AMR 2022, and 73.9% (193/261) by etiological analysis (81 pure and 112 mixed AMR). Detailed clinical evaluation attributed glomerulitis to sensitization in 38.0%, 10.3% from early ischemic DGF, nonadherence in 21.4%, or iatrogenic under immunosuppression in 30.3%.^30^ Glomerulitis scores were indistinguishable by etiology; however, Banff ptc, cg, MVI, cg, C4d_ptc_, C4d_glom_ scores, and DSA were significantly greater with AMR versus TCMR glomerulitis (Supplementary Table S4).

### Glomerulitis and Histological Antibody Markers

**Figure 3:**
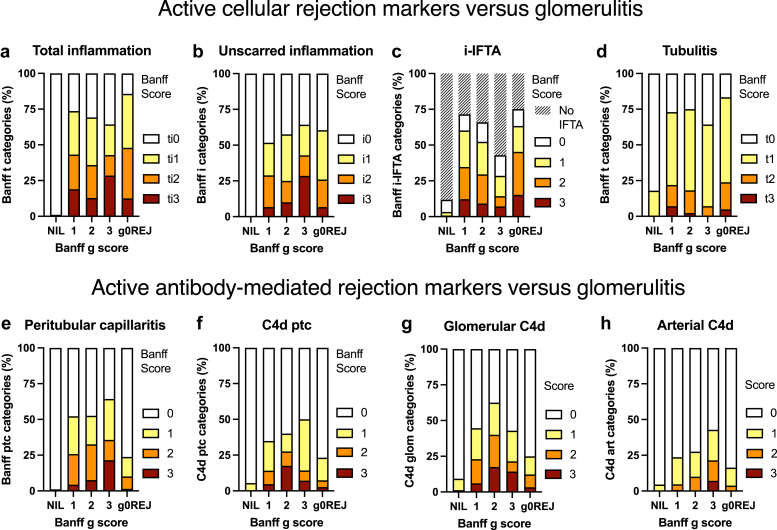
Histological associations with glomerulitis. Stack bars of the proportions of glomerulitis cases, subdivided by intensity of glomerular inflammation (Banff g score) and compared with normal control samples (NIL, *n* = 1442, left bars) and all-cause rejection without glomerulitis (g0REJ, *n* = 612 samples, right bars) as the positive comparator. The top register includes cellular rejection markers, including total and interstitial inflammation, i-IFTA marking chronic active T-cell–mediated rejection, and tubulits, which are all increased and comparable with g0REJ tissues. The bottom register includes histological markers of antibody-mediated rejection, including peritubular capillary inflammation (Banff ptc) and C4d staining in ptc, glomerular capillary loops, and muscular arteries using immunoperoxidase, which was increased in glomerulitis compared with g0REJ. C4d, complement degradation split-product 4d; g0REJ, all-cause rejection without glomerulitis (positive comparator); i-IFTA, inflammation within interstitial fibrosis and tubular atrophy; NIL, normal control samples.

Banff g score correlated Banff ptc (rho = 0.348, *P* < 0.001), cg (rho = 0.284, *P* < 0.001), mm (rho = 0.223, *P* < 0.001), i (rho = 0.240, *P* < 0.001), ti (rho = 0.214, *P* < 0.001), t (rho = 0.174, *P* < 0.001), v (rho = 0.152, *P* < 0.001), C4d_ptc_ (rho = 0.214, *P* < 0.001), C4d_glom_ (rho = 0.245, *P* < 0.001), C4d_art_ (rho = 0.180, *P* < 0.001), and DSA MFI (*r* = 0.198, *P* < 0.001, *n* = 3603). Binomial univariable generalized estimating equation restricted to histology found that glomerulitis was predicted by Banff ptc, ti, i, t, v, cg, mm, C4d scores, and DSA+ (all *P* < 0.001). Interstitial inflammation scores associated with glomerulitis were comparable with g0REJ (*n* = 612 from 351 kidneys without glomerulitis) but exceeded normal controls (*P* < 0.001, *n* = 1442 samples from 285 kidneys). Pathological AMR histology markers were increased in glomerulitis tissues compared with control kidneys (Figure 3, Supplementary Table S3).

**Figure 4:**
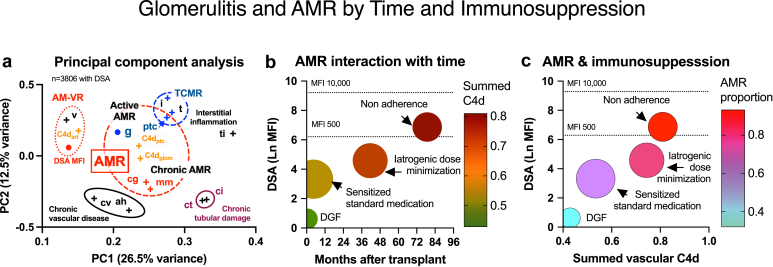
Relationships of glomerulitis to histology, antibody, and underimmunosuppression. (a) Principal component analysis co-localized glomerulitis (Banff g) to some active histological markers of AMR (Banff ptc, C4d_ptc_, and C4d_glom_). Chronic structural glomerulopathy (Banff cg, mm) and interstitial inflammation (i and t) were proximate, but distinct from tubulointerstitial scarring (ci and ct), chronic vasculopathy and arteriolar hyalinosis (cv and ah), and vascular AMR cluster (DSA, C4d_art_, and v). (b) Interaction plot of markers of AMR (DSA MFI, y-axis) and summated tissue C4d+ scores (C4d_ptc_ + C4d_glom_ + C4d_art_, red color) against posttransplant time (x-axis) and immunosuppression. Early sensitized patients on protocol medication and kidneys with primary DGF showed glomerulitis with low DSA and C4d values from T-cell–mediated rejection, compared with later underimmunosuppression from iatrogenic and patient nonadherence had higher MFI and stronger C4d+ tissue binding (darker red) from AMR. (c) Interaction plot of the relationship of glomerulitis with immunosuppression category and AMR. Glomerulitis from early DGF was unrelated to AMR (absent DSA and C4d), which compared with iatrogenic immunosuppression reduction and nonadherence with AMR markers appeared greater than DSA strength (y-axis), C4d+ tissue binding (x-axis), and AMR classification by root cause analysis. Dot sizes are relative biopsy numbers. Total, interstitial, tubular, arterial, and peritubular capillary inflammation are Banff ti, i, t, v, ptc, chronic arterial and arteriolar lesions are Banff cv and ah, and chronic fibrosis and tubular atrophy are Banff ci and ct. AMR, antibody-mediated rejection; C4d, complement degradation split-product 4d; DGF, delayed graft function; DSA, donor-specific antibody: MFI, median fluorescence intensity.

Banff g score histological associations were confirmed by ordinal regression for Banff ptc, cg, i, C4d_glom_, and C4d_art_ scores, although C4d_ptc_+ and DSA+ lost significance on multivariable analysis (data not shown). Cohort principal component analysis colocalized glomerulitis microvascular AMR cluster (Banff ptc, cg, mm, C4d_ptc_, C4d_glom_) and adjacent to interstitial inflammation (Banff i and t, *n* = 3806, Figure 4a). In glomerulitis defined by light microscopy, glomerular endothelium was ultrastructurally abnormal in 68.5% (including mild cg0e), with 51.0% showing Banff ≥ cg1a and 40.1% demonstrating peritubular capillary multi-lamination ≥ 4 (*n* = 2211 samples from 1081 patients with DSA results).

### Glomerulitis Stratified by DSA Results

**Figure 5:**
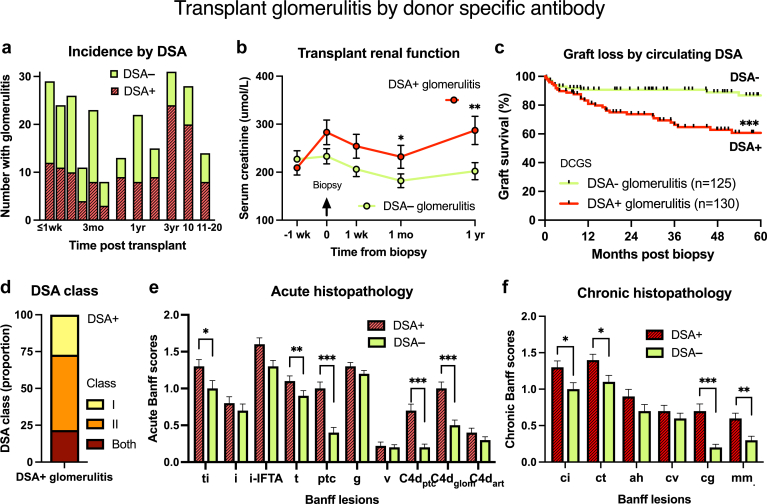
Clinical outcomes and pathological phenotype of glomerulitis by circulating DSA. (a) The incidence of DSA+ glomerulitis increased with posttransplant time. Many DSA− glomerulitis presented early after transplantation. (b) Sustained serum creatinine improvements followed treatment of DSA− glomerulitis compared with DSA+ glomerulitis, which experienced late functional decline. (c) Death-censored graft loss was greater in DSA+ glomerulitis versus kidneys without DSA. (d) Class I and II DSA frequencies in DSA+ glomerulitis. (e) Kidneys with DSA+ glomerulitis showed increased active tubular and peritubular capillary inflammation (Banff t and ptc), peritubular and glomerular C4d+ (C4d_ptc_ and C4d_glom_), chronic transplant glomerulopathy (cg and mm lesions) as markers of chronic active AMR with tubulointerstitial scarring (ci and ct) compared with DSA− cases (Panel f). Key: Mean±SEM. ∗*P* < 0.05, ∗∗*P* < 0.01, ∗∗∗*P* < 0.001 versus DSA− glomerulitis. C4d, complement degradation split-product 4d; DCGS, death-censored graft survival; DSA, donor-specific antibody.

Subanalysis found that DSA+ glomerulitis (50.9%, *n* = 130/255) was associated with younger recipient age, early AMR or vascular rejection, previous pulse corticosteroid treatment, and later biopsy presentation (12.0 months median) than DSA− cases (Figure 5). Clinical correlation revealed sensitization in 31.5%, iatrogenic dose reduction in 35.4%, and nonadherence in 31.5% (Figures 4b and c). DSA+ glomerulitis kidneys expressed greater Banff C4d_ptc_, C4d_glom_, cg, mm, ptc, and t scores, proteinuria, MFI, and class II DSA, reflecting chronic-active AMR (versus DSA−, Table 1, Supplementary Table S5). AMR was diagnosed in 80.0% using Banff 2019, 100.0% by Banff 2022 (including probable), and 91.5% using forensic etiological analysis (52.3% were mixed AMR/TCMR). Glomerular endothelium was abnormal using electron microscopy in 82.3% of DSA+ glomerulitis (vs. 50.8% DSA−). Treatment included pulse corticosteroids (63.1%), antithymocyte globulin (24.6%), plasma exchanage/i.v.Ig (24.6%), and/or upscaled medications (51.5%). Serum creatinine transiently stabilized, but often deteriorated with greater graft loss (*P* < 0.001 vs. DSA−, Figure 5b and c).

**Table 1:** Etiological classification of glomerulitis

**DSA category**	DSA+	DSA−	*P*-value
Biopsies (*n*)	130	125	
Kidneys	96	103	
Banff schema aligned diagnosis			
Banff 2019 AMR *n* (%)	104 (80.0)	43 (34.4)	<0.001
Banff 2022 AMR *n* (%)	130 (100.0)	25 (20.0)	<0.001
Banff 2022 TCMR *n* (%)	71 (54.6)	54 (43.2)	0.069
Root cause analysis: (*n*)	130	122	
Ischemia PMN *n* (%)	1 (0.8)	6 (4.9)	0.046
Pure TCMR *n* (%)	10 (7.7)	53 (43.3)	<0.001
Pure AMR *n* (%)	51 (39.2)	28 (23.0)	0.005
Mixed AMR/TCMR *n* (%)	68 (52.3)	35 (28.7)	0.034
Any attributed AMR *n* (%)	119 (91.5)	63 (51.6)	<0.001

C4d_ptc_ category	C4d_ptc_−	C4d_ptc_+	*P*-value
Biopsies (*n*)	188	83	
Kidneys	158	68	
Banff schema aligned diagnosis			
Banff 2019 AMR *n* (%)	73 (38.8)	83 (100.0)	<0.001
Banff 2022 AMR *n* (%)	79 (42.0)	83 (100.0)	<0.001
Banff 2022 TCMR *n* (%)	75 (39.9)	57 (68.7)	<0.001
Root cause analysis (*n*)	185	83	
Ischemia PMN *n* (%)	7 (3.8)	0 (0.0)	<0.001
Pure TCMR *n* (%)	68 (36.8)	0 (0.0)	<0.001
Pure AMR *n* (%)	52 (28.1)	29 (34.9)	0.261
Mixed AMR/TCMR *n* (%)	58 (31.3)	83 (100.0)	<0.001

Glomerulitis category	Isolated	Inflamed	*P*-value
Biopsies (*n*)	55	216	
Kidneys	51	167	
Banff schema aligned diagnosis			
Banff 2019 AMR *n* (%)	9 (16.4)	147 (68.1)	<0.001
Banff 2022 AMR *n* (%)	23 (41.8)	139 (64.4)	0.002
Banff 2022 TCMR *n* (%)	1 (1.8)	131 (60.6)	<0.001
Root cause analysis: (*n*)	54	214	
Ischemia PMN *n* (%)	6 (11.1)	1 (0.5)	<0.001
Pure TCMR *n* (%)	28 (51.9)	40 (18.7)	<0.001
Pure AMR *n* (%)	19 (35.2)	62 (29.0)	0.375
Mixed AMR/TCMR *n* (%)	1 (1.9)	111 (51.9)	<0.001

AMR, antibody-mediated rejection; DSA, donor-specific antibody; PMN, polymorphonuclear neutrophil (leukocyte); TCMR, T-cell–mediated rejection.

Biopsy prevalence of glomerulitis was 6.3% and 15.4% of kidneys.

In contrast, DSA− glomerulitis (*n* = 125, 46.1%) presented earlier (median 3 months) with lower tissue C4d_ptc_+, C4d_glom_+, and chronic glomerulopathy scores (*P* < 0.001 vs. DSA+) and 62.4% were presensitized. Treatment was corticosteroids (56.0%), antithymocyte globulin (16.8%), and upscaled medications (33.6%). Banff 2019 diagnosed AMR in 34.4% and 20.0% using Banff 2022 AMR criteria (including probable). Banff TCMR occurred in 43.2%. Etiological classification assigned 43.3% pure TCMR, 28.7% mixed AMR/TCMR, 23.0% pure AMR, and 4.9% ischemic neutrophilic glomerulitis. Graft survival of DSA− glomerulitis paralleled normal kidneys without rejection (*P* = 0.157 differences).

### C4d_ptc_ Negative Glomerulitis

**Figure 6:**
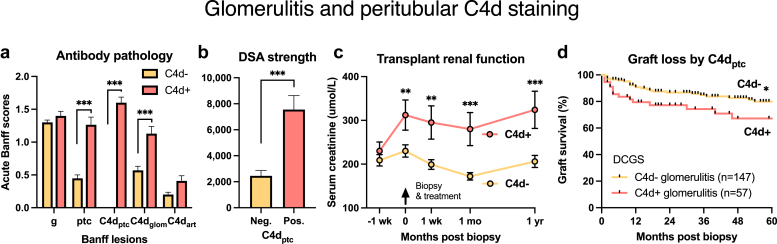
Glomerulitis by C4d_ptc_ immunohistochemistry. (a) C4d_ptc_+ glomerulitis was associated with increased peritubular capillary inflammation (Banff ptc) and glomerular C4d (C4d_glom_), DSA strength by median fluorescence intensity (MFI) (Panel b), renal dysfunction impairment (Panel c) and death-censored graft loss versus C4d_ptc_- glomerulitis (Panel d). C4d, complement degradation split-product 4d; DCGS, death-censored graft survival; DSA, donor-specific antibody.

Glomerulitis without C4d_ptc_ (69.4% prevalence, 188/271), presented earlier with lower serum creatinine, acute inflammatory histology, C4d_glom_+, and DSA+ compared with C4d_ptc_+ glomerulitis (Figure 6a and b, Table 1, Suppplementary Table S6). DSA+ was found in 44.1%, C4d_glom_+ in 33.5%, abnormal endothelium in 59.4%, and peritubular capillary multi-lamination ≥ 4 in 35.4%. Banff diagnosed AMR in 38.8% (using 2019 MVI ≥ 2 and DSA+) and 42.0% (Banff 2022 including probable), with TCMR present in 39.9%. Root cause etiological analysis (including CD3+ glomerular immunophenotyping) classified 36.8% as pure T-cell glomerulitis, 28.1% as pure AMR, and 31.3% mixed AMR/TCMR pathophysiology. Overall, AMR was attributed to 59.3% of C4d_ptc_− glomerulitis using etiological analysis, of which 73.4% were treated. Renal functional recovery and graft survival of C4d_ptc_− glomerulitis was better than C4d_ptc_+ kidneys (Figure 6c and d).

Subanalysis of DSA−/C4d_ptc_− “double negative” glomerulitis (*n* = 100 cases) revealed early sensitization in 45.0%, DGF or ischemia in 20.0%, and iatrogenic underimmunosuppression in 26.0%. Banff diagnosed TCMR in 36.0%, AMR in 18.0% using 2019 but 0% using the 2022 criteria (i.e., leaving 82.0% unclassified). Etiological classification attributed 54.6% to cellular rejection, 20.6% pure AMR, and 18.6% as mixed AMR/TCMR summating to 39.2% with some evidence of antibody causation (details are in Table 2 and Supplementary Table S7). Glomerular capillary endothelium was abnormal in 42.5% by electron microscopy (20/47 examined). Compared with AMR cases (DSA or C4d_ptc_+, *n* = 155), “double negative” glomerulitis demonstrated lower tubulo-interstitial, microvascular, and arterial inflammation score with less chronic scarring and glomerulopathy.

**Table 2:** Double-negative glomerulitis without C4d_ptc_ or DSA: key differences

Category	DSA−	DSA+ or	*P*-value
	C4d_ptc_−	C4d_ptc_+	
LM /EM biopsies (*n*)	100/47	155/93	
Time (mos)	17.4 ± 40.8	41.7 ± 60.0	<0.001
Light microscopy			
Banff g score	1.2 ± 0.5	1.3 ± 0.6	0.034
Banff ptc score	0.3 ± 0.7	0.9 ± 1.0	<0.001
Banff i score	0.6 ± 0.9	0.9 ± 0.9	0.018
Banff ti score	0.8 ± 0.9	1.4 ± 1.1	<0.001
Banff t score	0.8 ± 0.8	1.2 ± 0.8	<0.001
Banff ci score	0.9 ± 1.0	1.3 ± 1.0	<0.001
Banff cg score	0.2 ± 0.5	0.6 ± 1.0	<0.001
Any C4d_ptc_*n* (%)	0 (0)	76 (49.0)	<0.001
Any C4d_glom_*n* (%)	29 (29.0)	82 (52.9)	<0.001
Glomerular capillary endothelial ultrastructure			
(*n*, % abnormal)	20 (42.5)	76 (81.7)	<0.001
Banff cg ≥ 1a (*n*)	13 (27.6)	59 (63.4%)	<0.001
Podocyte effacement (score)	1.0 ± 0.6	1.3 ± 0.9	0.031
PTC-ML (maximal layers)	2.7 ± 1.5	4.3 ± 2.6	<0.001
Banff schema aligned diagnosis			
Banff 2022 TCMR *n* (%)	36 (36.0)	89 (57.4)	<0.001
Banff 2019 AMR *n* (%)	0 (0)	114 (73.5)	<0.001
Banff 2022 AMR (including probable) *n* (%)	0 (0)	100 (100.0)	<0.001
Root cause analysis: (*n*)	97	155	
Ischemia PMN *n* (%)	6 (6.2)	1 (0.6)	<0.001
Pure TCMR *n* (%)	53 (54.6)	10 (6.5)	<0.001
Pure AMR *n* (%)	20 (20.6)	59 (38.1)	0.002
Mixed AMR/TCMR *n* (%)	18 (18.6)	85 (54.8)	<0.001
Any attributed AMR *n* (%)	38 (39.2)	144 (92.9)	<0.001

AMR, antibody-mediated rejection; C4d, complement degradation split-product 4d by immunohistochemistry of peritubular (ptc) or glomerular capillaries (glom); DSA, donor-specific antibody; EM, electron microscopy; LM, light microscopy; PMN, polymorphonuclear neutrophil leukocyte. PTC-ML, peritubular capillary multi-lamination; TCMR, T cell mediated rejection.

Glomerulitis without DSA or C4d_ptc_− was compared with AMR glomerulitis with either C4d_ptc_+ or DSA+. Detailed data in Supplementary Table S7. Mean ± SD, (*n*, %).

### Isolated Glomerulitis

**Figure 7:**
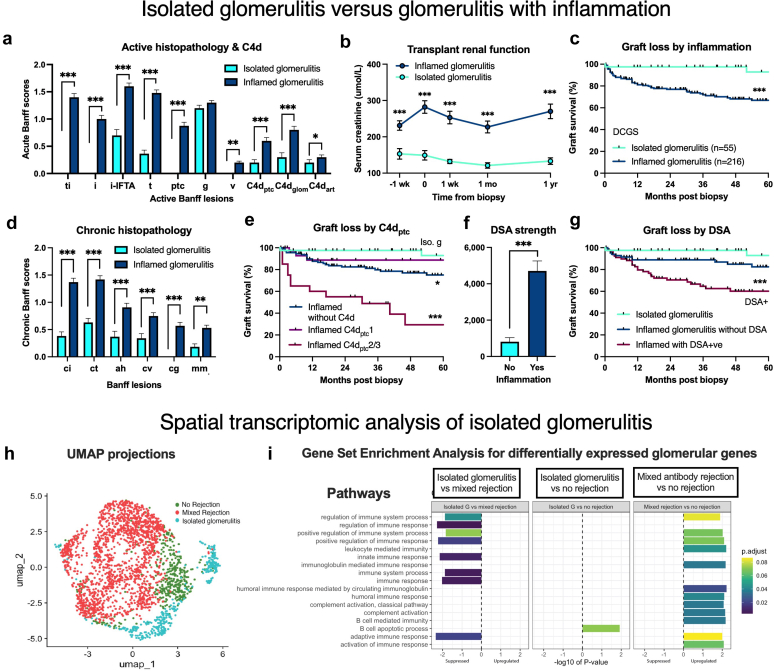
Isolated glomerulitis versus glomerulitis with inflammation. (a) Isolated glomerulitis was characterized by minimal or absent inflammation in the tubulointerstitial compartment (Banff ti, i, and t) and peritubular capillaries (Banff ptc) with lesser C4d staining in peritubular and glomerular capillaries and muscular arteries (C4d_ptc_, C4d_glom_, C4d_art_). (b) Renal dysfunction, death-censored graft loss (Panel c), and chronic pathological damage (Panel d) were minimal with isolated glomerulitis. (e) Graft loss with inflamed glomerulitis was greater, moderate, or severe C4d_ptc_ staining. (f) The median fluorescence intensity of DSA was greater with inflamed glomerulitis, as was death-censored graft loss with DSA+ cases compared with isolated glomerulitis (Panel g). (h) UMAP projections of spatial transcriptomics stratified by sample group with separation of isolated glomerulitis (blue, 5 glomeruli) from mixed antibody-mediated rejection (red, *n* = 12) and no rejection (*n* = 7 negative control glomeruli). (i) Gene set enrichment analysis for differentially expressed genes with adjusted *P* < 0.1 and log_2_-FC > 0. When isolated glomerulitis was compared with negative control glomeruli without rejection, the only differentially expressed gene was modest upregulation of B-cell apoptotic process (adjusted *P* = 0.072). Chronic mixed rejection was associated with upregulation of innate, leukocyte and adaptive pathways versus both isolated glomerulitis and negative controls. Key: Mean ± SEM. Key: ∗*P* < 0.05, ∗∗*P* < 0.01, ∗∗∗*P* < 0.001. DCGS, death-censored graft survival; DSA, donor-specific antibody; i-IFTA, inflammation within interstitial fibrosis and tubular atrophy.

Glomerulitis without associated vascular or interstitial inflammation (Banff ti, i, ptc, v, and cg all absent) occurred in 20.3% (55/271) and was diagnosed by surveillance sampling in 70.9%. Compared with glomerulitis with inflammation, isolated glomerulitis presented earlier with milder acute and chronic pathology, C4d, and DSA (Figure 7a and d, Supplementary Table S8). Infrequent AMR markers included C4d_ptc_+ (14.5%, 47/55), C4d_glom_+ (20.0%), and DSA+ (41.7%, Figure 7f). Banff 2019 diagnosed AMR in 16.4% (often g2MVI with DSA+ and/or C4d_ptc_+), Banff 2022 called 41.8% AMR (including probable), with Banff TCMR recognized in only 1.8% (Table 1). Most were unclassifiable by any Banff schema. Etiological analysis including immunophenotyping found isolated CD3+ TCMR glomerulitis in 51.9%, and attributable AMR features in 35.2% (C4d_ptc_+, C4d_glom_+, and/or DSA+ criteria including previous antibody). Glomerular endothelium was ultrastructurally abnormal in 16.4% of isolated glomerulitis (vs. 75.4% for inflamed glomerulitis, *P* < 0.001).

Spatial transcriptomics analysis showed separation by lesion type for isolated glomerulitis using UMAP projection (Figure 7h). Gene set enrichment analysis found a negligible inflammatory phenotype with minor upregulation of B-cell apoptotic process (adjusted *P* = 0.072) in isolated glomerulitis compared with negative controls (no rejection, Figure 7i, Supplementary Table S9). Unsurprisingly, chronic mixed AMR comparator tissues were characterized by upregulated innate, leukocyte, and adaptive pathways versus either isolated glomerulitis or negative controls.

Renal dysfunction occurred in 27.3% of isolated glomerulitis and improved following treatment (Figure 7c). Only 40.0% were treated using pulse corticosteroids (23.6%) and/or upscaled therapy (16.4%). Most were left untreated and glomerulitis belatedly resolved by sequential histology. Graft survival was indistinguishable from normal controls (*P* = 0.778, Figure 7c). The increased graft loss with inflamed glomerulitis was influenced by C4d_ptc_+ and DSA+ (Figure 7e and f).

### Diagnostic Test Performance of Glomerulitis for AMR

AMR was diagnosed in 57.6% (156/271) of glomerulitis versus 2.0% of kidneys without glomerulitis (89/4029, *P* < 0.001). Against Banff AMR 2019 test reference, respective sensitivity and specificity for glomerulitis (Banff g ≥ 1) for AMR diagnosis were 60.5% and 96.3%, with positive and negative predictive values being 44.7% and 98.0%, respectively (*n* = 4300). Using Banff 2022 AMR reference standard, sensitivity and specificity were 47.5% and 97.3%, respectively; predictive value was 59.8%, and negative predictive value was 93.3%. The agreement between Banff AMR 2019 and 2022 was Cohen’s kappa of 0.640 (95% confidence interval: 0.546–0.735). Against our etiological AMR classification, the respective kappa was 0.702 (95% confidence interval: 0.617–0.789) against 2019 and 0.564 (95% confidence interval: 0.464–0.667) for Banff AMR 2022 (including probable: overcalling glomerulitis with low-level DSA).

### Graft Failure With Glomerulitis

Death-censored graft survival was calculated in 204 unique kidneys with glomerulitis (follow-up of 39.4 months, interquartile range: 14.0–71.5). Respective 1, 5, and 10-year death-censored graft survival rates were 87.1%, 76.2%, and 68.2% for glomerulitis kidneys; 93.6%, 80.0%, and 70.9% for g0REJ (*n* = 351); and 99.2%, 91.8%, and 81.8% for normal control kidneys (*n* = 285, *P* < 0.001). One-, 5- and 10-year patient survivals were 97.4%, 82.8%, and 68.1%, respectively, for recipients with glomerulitis.

**Figure 8:**
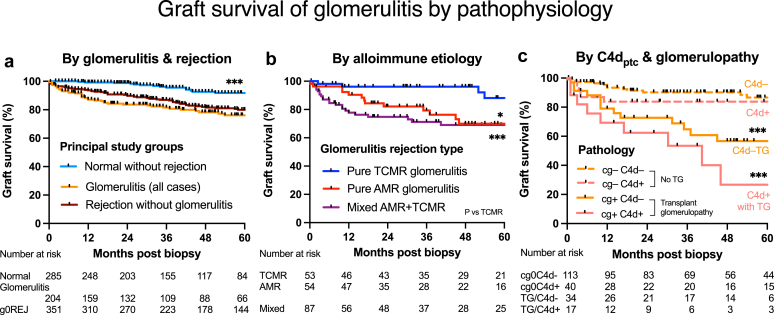
Graft survival of glomerulitis stratified by scenario. (a) Death-censored graft survival of the principal study groups including glomerulitis (*n* = 204 unique kidneys) was decreased compared with normal control kidneys (*n* = 285), but comparable with g0REJ, (*n* = 351). (b) Graft loss was worst with glomerulitis within mixed rejection, intermediate for pure AMR, and best for TCMR glomerulitis. (c) Factorial analysis of death-censored graft survival demonstrated poor outcomes with chronic TG (Banff cg^3^ 1 vs. none), which were amplified by C4d_ptc_+ representing endothelial donor-specific antibody binding to tissue and local complement activation. Key: ∗*P* < 0.05, ∗∗*P* < 0.01, ∗∗∗*P* < 0.001. AMR, antibody-mediated rejection; C4d, complement degradation split-product 4d; g0REJ, all-cause rejection without glomerulitis (positive comparator); TCMR, T-cell–mediated rejection; TG, transplant glomerulopathy.

Graft survival was considered from 3 perspectives. Population death-censored graft survival for kidneys with glomerulitis or g0REJ were both reduced versus normal kidney controls (*P* < 0.001, Figure 8a). For glomerulitis kidneys, Kaplan-Meier survival was reduced by DSA+ (logrank 8.185, *P* = 0.004, Figures 5c and 7g), C4d_ptc_+ (logrank 5.733, *P* = 0.017, Figures 6d and 7e), and C4d_glom_+ (logrank 11.954, *P* < 0.001). Compared with TCMR glomerulitis, graft survival was reduced with pure AMR glomerulitis (logrank 5.659, *P* = 0.017) and mixed AMR/TCMR phenotypes (logrank 10.950, *P* < 0.001, Figure 8b).

Univariable histological predictors of death-censored graft survival in glomerulitis kidneys included Banff ptc, MVI, C4d_ptc_, C4d_glom_, cg, mm, ci, ct, cv scores, SV40+, and DSA+ using Cox regression. Banff g score and dichotomized glomerulitis had no influence on survival. Multivariable regression found Banff C4d_ptc_, ci, and cg scores independently predicted graft loss when controlled for DSA strength as MFI (Figure 8c, Table 3, Supplementary Table S10). An alternative mixed clinicopathological model found renal dysfunction, proteinuria, Banff ci score, and DSA strength predicted graft failure.

**Table 3:** Multivariable predictors of allograft failure in glomerulitis

	HR	95% CI	*P*-value
Histopathological restricted model 1 including DSA			
Banff ci score	2.364	1.691–3.305	< 0.001
Banff cg score	1.446	1.064–1.965	0.018
C4d_ptc_ score	1.441	1.078–1.927	0.014
DSA ln (MFI+1)	1.105	1.010–1.209	0.030
Parsimonious clinical and pathological mixed model 2			
S. creatinine (umol/l)	1.002	1.001–1.002	< 0.001
Urinary albumin/creatinine (mg/mmol)	1.004	1.002–1.006	< 0.001
DSA ln (MFI+1)	1.129	1.025–1.243	0.014
Banff ci score	2.264	1.793–3.892	< 0.001

Banff cg, chronic glomerulopathy; Banff ci, chronic interstitial fibrosis; C4d_ptc_, C4d staining of peritubular capillaries; CI, confidence interval; DSA, donor-specific antibody; HR, hazard ratio; MFI, median fluorescence intensity.

Predictors of death-censored allograft failure in kidneys with glomerulitis from its first occurrence in a unique kidney (*n* = 204) using multivariable Cox regression restricted to key pathological lesions and DSA. Chronic active antibody-mediated rejection pathophysiology (C4d_ptc_+, Banff cg, and DSA “strength”) and interstitial scarring (ci fibrosis score reflecting nephron loss), renal dysfunction (serum creatinine concentration), and proteinuria (urinary albumin-to-creatinine ratio, mg/mmol) were important independent determinants of kidney survival. Ordinal and dichotomized glomerulitis scores were not significant in either multivariable model.

## Discussion

This large and detailed study reveals the diverse clinical and pathological spectrum of transplant glomerulitis within 15.4% of kidneys and 6.3% of all biopsy tissues. Epidemiological analysis found that female recipients, living donation, early AMR, TCMR, and previous corticosteroid rejection treatment were risk factors. Patient-centered evaluation revealed nonadherence in 21.4% and iatrogenic underdosing in 30.3%, predominantly for infection. Rejection was the primary diagnosis in the majority, with Banff 2019 and 2022 AMR diagnosed in 57.6% and 59.8%, respectively; and/or TCMR in 49.2% of glomerulitis. Many incomplete phenotypes were unclassifiable using orthodox Banff schema. Correspondingly, etiological classification used comprehensive clinical, serological, and pathological evaluation (including glomerular capillary endothelial changes or nonconventional C4d_glom_+ binding, immunophenotyping, historical DSA+, and previous AMR episodes) to assign likely immunopathology. Glomerulitis appeared within surveillance tissues from ischemic kidneys with DGF, inflammatory BK virus allograft nephropathy, nonspecific scarring, and rarely in otherwise normal transplant tissues. Dichotomization using DSA+ and C4d_ptc_+ status illuminated interplay between AMR and antibody-independent glomerular inflammation.

DSA+ glomerulitis was histologically typified by active inflammation, C4d_ptc_+, C4d_glom_+, transplant glomerulopathy, and chronic interstitial fibrosis in kidneys with inferior survival than DSA− glomerulitis. AMR was diagnosed in 80.0% using Banff 2019, 100.0% by Banff 2022, and 91.5% by etiological classification. Mixed AMR with interstitial TCMR occurred in 52.3%. Glomerular inflammation correlated with DSA strength, C4d+ score, and histological markers of AMR. Glomerulitis kidneys classified by DSA+ or AMR etiology failed more frequently than normal controls, mirroring other reports of histological AMR.^31^, ^32^, ^33^, ^34^ In contrast, DSA− glomerulitis was typically negative for C4d_ptc_ and C4d_glom_, and expressed a milder AMR phenotype in 34.4% and 20.0% using Banff 2019/22, and 51.6% by etiological classification including C4d_glom_+. Adsorption of circulating antibody, technical failure, non-HLA antibodies, subthreshold or shared-eplet DSA are explanations for missed AMR diagnosis.^16^^,^^35^ Pure interstitial Banff TCMR was accompanied by CD3+ glomerulitis in 43.3% of DSA− glomerulitis, with mixed AMR/TCMR in 28.7%. Graft survival was comparable with normal controls. C4d_ptc_− glomerulitis was common (69.4%) and equally mediated by TCMR, mixed AMR/TCMR, and mild pure AMR: with superior outcomes compared with C4d_ptc_+ kidneys. Hence, glomerulitis demonstrating any evidence of antibody including DSA+, C4d+, or AMR histology was followed by adverse outcomes.

Approximately half of DSA− and/or C4d_ptc_− glomerulitis cases were independent of alloantibody and instead mediated by T-cell and/or innate microvascular inflammation. Early mononuclear or neutrophilic glomerulitis was common (20.7%) in surveillance DGF samples and typically mild (g = 1), DSA−, and C4d_ptc_−, rapidly resolving on repeat tissue sampling concurrent with functional recovery. Ischemic endothelial cells release or upregulate damage-associated molecular pattern molecules, integrins (ICAM and VCAM), MCP-1, P-selectin, and von Willebrand factor, which cause local glomerular retention of passaging leukocytes by “sticky endothelium” constituting nonalloimmune pathophysiology.^12^^,^^36^^,^^37^ Alternatively, antibody-independent innate inflammation encompassing NK cell activation (via KIR receptors as “missing self”), direct monocyte allorecognition, or primary T-cell activation toward mismatched HLA endothelial molecules are possible alloimmune explanations.^38^^,^^39^

DSA− CD3+ glomerulitis seen in isolation or within interstitial TCMR (“g-i-t” pattern) was relatively common early after transplantation, clinically mild, and generally lacked EOL. Immunophenotyping of early CD3+ T-cell glomerulitis from acute C4d_ptc_− interstitial TCMR has been contrasted with later DSA+ AMR glomerulitis dominated by CD68+ monocytes and C4d_ptc_+ in other studies.^40^, ^41^, ^42^, ^43^, ^44^, ^45^ Early MVI and glomerulitis often appear antibody-independent using DSA, C4d_ptc_, or molecular AMR classifiers.^46^^,^^47^ Glomerulitis is not an orthodox diagnostic feature of TCMR and occurred in only 6.7% of Banff confirmed diagnoses. However, when glomerulitis cases were aggregated, TCMR with concurrent glomerulitis was observed in 49.2% as immunological “spillover” (top panel Figure 2). The literature reports glomerulitis in 31.4% to 56%.^3^^,^^12^^,^^44^^,^^48^ A systematic review found that glomerulitis (and MVI) in active TCMR was less prevalent and severe than AMR glomerulitis.^49^ Multiomic profiling of C4d_ptc_−/DSA− transplant glomerulopathy demonstrated novel T-cell dominant subclasses distinct from glomerular macrophages and NK cells in C4d_ptc_+/DSA+ chronic AMR.^19^ Glomerulitis is simply a morphological description of intracapillary glomerular leukocytes rather than a specific etiological diagnosis of AMR. Known differential diagnoses include recurrent glomerulonephritis, viral inflammation (e.g., cytomegalovirus and BK virus), and rare complement-associated diseases (e.g., atypical hemolytic uremic syndrome and thrombotic microangiopathy) were our study exclusions. Therefore, not all glomerulitis cases are caused by AMR: a substantial minority of antibody-independent episodes were driven by CD3+ TCMR or innate mononuclear MVI.

These results question the ability of the glomerulitis lesion to correctly classify AMR within any diagnostic schema. Although glomerulitis showed an excellent 97.3% specificity using Banff 2022 AMR criteria, its suboptimal 59.8% positive predictive value guarantees some misclassification, which increases when AMR prevalence falls and false positive rates naturally increase.^36^ Both MVI ≥ 2 (until Banff 2019) and ptc ≥ 1 are disqualified as AMR diagnostic criteria with interstitial TCMR unless glomerulitis is also present. Banff 2013 originally considered C4d_ptc_−/DSA− glomerulitis below the AMR diagnostic threshold,^4^ whereas Banff 2022 repositioned DSA−/C4d_ptc_− MVI ≥ 2 into an indeterminant category. Glomerulitis is not a pathognomonic feature of AMR with privileged rights of adjudication. AMR independent mechanisms apply to ischemia-dependent inflammation, C4d_ptc_−, C4d_glom_−, and CD3+ T-cell glomerulitis. Instead, we advocate comprehensive pathological and serological evaluation of incomplete phenotypes, including DSA−/C4d_ptc_− glomerulitis by CD3/68 and C4d_glom_ immunohistochemistry, specialist serological evaluation for subthreshold or shared-eplet DSA, and molecular transcriptomics if available.^19^^,^^22^^,^^30^^,^^46^^,^^50^

Many mild glomerulitis cases were incomplete phenotypes and unclassifiable using the current Banff schema. Isolated glomerulitis without any arterial, peritubular capillary, or interstitial inflammation was unexpectedly common in 20.3% and typically discovered by surveillance sampling. Banff 2019 diagnosed AMR diagnosis in only 16.4% (using DSA+ and C4d_ptc_+ criteria); Banff 2022 (including probable AMR with low-level DSA+) called 41.8% AMR, and comprehensive etiological assessment (including C4d_glom_+) classified 35.2% as AMR. Immunophenotyping revealed isolated CD3+ T-cell glomerulitis in 51.9%. Subclinical isolated g1 in stable kidneys was often left untreated and mostly histologically resolved. Pulse corticosteroid treatment was reserved for allograft dysfunction, DSA+, C4d+ (including C4d_glom_+), and recurrent or diffuse g2/3 with EOL.

Studies of “isolated glomerulitis” (differently defined as absent DSA/C4d_ptc_) in stable 3-month protocol samples report excellent outcomes indistinguishable from matched negative controls in 1 study^51^ but inferior results for dysfunctional kidneys equivalent to TCMR or AMR comparators.^48^ Study interpretation is constrained by the use of historical diffuse > 50% C4d_ptc_+ thresholds and outdated DSA technology. Bulk molecular diagnostics identified AMR in only 6% of isolated glomerulitis.^46^ Our spatial transcriptomic phenotype of DSA− isolated glomerulitis showed minimal parenchymal disturbance comparable to nonrejection glomeruli. Given the favorable clinical features and excellent outcomes irrespective of pathophysiology; isolated glomerulitis should be considered a partial phenotype of mild immune activation, analogous to “isolated v” or “isolated tubulitis”.^23^^,^^52^^,^^53^

Transplant kidneys with glomerulitis were generally characterized by a greater burden of inflammation and chronic tubular damage and failed more frequently than normal controls.^12^^,^^40^, ^41^, ^42^^,^^44^^,^^48^^,^^54^^,^^55^ Graft loss was predicted by interstitial fibrosis (nephron loss), transplant glomerulopathy and C4d_ptc_+ (denoting chronic and active AMR, respectively), and DSA strength; but not by glomerulitis score using multivariable histology analysis.^56^ Chronic damage markers better predicted graft survival than acute inflammatory lesions in this and another study of late AMR^57^; however, intense CD68+ glomerulitis forecast deterioration in another report.^42^ The dismal survival of mixed AMR glomerulitis likely represents a broader alloimmune response than either pure AMR or TCMR alone. However, not all expressions of glomerulitis were harmful: early ischemic innate inflammation, DSA−, C4d_ptc_−, and isolated glomerulitis all appeared relatively benign, transient, and easily treated with good outcomes.

Study strengths include a large, well-phenotyped cohort with individual patient-level data, C4d immunoperoxidase (including C4d_glom_), solid-phase DSA including DP/DQ, ultrastructural endothelial evaluation, spatial transcriptomics, and abundant rejection and normal control cases for statistical comparisons. We used the long-established Banff 1997 definition to avoid exclusion of mild g1 without EOL (otherwise inappropriately classified “normal” g0).^2^^,^^7^ Our single-center study used prospective data collection and constitutes the largest study of glomerulitis to-date. Follow-up was complete and missing data virtually absent. Vertical integration of multilayered data links histological glomerulitis to an individual recipient and their transplanted kidney: a holistic approach promoting accurate and personalized medicine.

In summary, though glomerulitis frequently signifies histological AMR, a substantial minority were driven by TCMR or innate cellular inflammation. Transplant glomerulitis expressed within a highly diverse spectrum of clinical and pathological phenotypes that ranges from mild, isolated, DSA−, and C4d_ptc_− glomerulitis with minimal allograft impact to severe DSA+, C4d_ptc_+, C4d_glom_+ AMR glomerulitis within scarred and inflamed kidneys from underimmunosuppression that foreshadows imminent graft failure. Diagnostic evaluation incorporating comprehensive clinical, serological, pathological, and molecular information allows for precise etiological classification for optimal patient-centered management.

## Disclosure

JSYL is a recipient of the National Health and Medical Research Council (NHMRC) Ideas Grant GNT2030303. XD received a Research Training Program Stipend, SC3227 from the University of Sydney. All the other authors declared no competing interests.

## Supplementary Material

Supplementary File (PDF) **Table S1.** STROBE guidelines for diagnostic studies: checklist. **Table S2.** Spatial transcriptomics methods. **Table S3.** Pathology of glomerulitis versus samples without glomerulitis. **Table S4.** Glomerulitis by dominant rejection pathology. **Table S5.** Pathology of glomerulitis by DSA detection. **Table S6.** Pathology of glomerulitis by C4d_ptc_ status. **Table S7.** Double-negative glomerulitis without C4d_ptc_ or DSA. **Table S8.** Isolated glomerulitis versus glomerulitis with inflammation. **Table S9.** Detailed spatial transcriptomics results. **Table S10.** Determinants of graft failure.

## Footnotes

Supplementary File (PDF)
**Table S1.** STROBE guidelines for diagnostic studies: checklist. **Table S2.** Spatial transcriptomics methods. **Table S3.** Pathology of glomerulitis versus samples without glomerulitis. **Table S4.** Glomerulitis by dominant rejection pathology. **Table S5.** Pathology of glomerulitis by DSA detection. **Table S6.** Pathology of glomerulitis by C4d_ptc_ status. **Table S7.** Double-negative glomerulitis without C4d_ptc_ or DSA. **Table S8.** Isolated glomerulitis versus glomerulitis with inflammation. **Table S9.** Detailed spatial transcriptomics results. **Table S10.** Determinants of graft failure.
