# Supplementary material for: The Diversity of Transplant Glomerulitis and its Relationship to Alloantibody
Source: Kidney Int Rep. 2026 Feb 25;11(5):106378. doi: 10.1016/j.ekir.2026.106378 (PMC13090548; doi:10.1016/j.ekir.2026.106378)
Supplement: Supplementary File (PDF) — Table S1. STROBE guidelines for diagnostic studies: checklist. Table S2. Spatial transcriptomics methods. Table S3. Pathology of glomerulitis versus samples without glomerulitis. Table S4. Glomerulitis by dominant rejection pathology. Table S5. Pathology of glomerulitis by DSA detection. Table S6. Pathology of glomerulitis by C4dptc status. Table S7. Double-negative glomerulitis without C4dptc or DSA. Table S8. Isolated glomerulitis versus glomerulitis with inflammation. Table S9. Detailed spatial transcriptomics results. Table S10. Determinants of graft failure. [file mmc1.pdf]

Supplementary Content

Table S1: STROBE guidelines for diagnostic studies: checklist.
Table S2: Spatial transcriptomics methods
Table S3: Pathology of glomerulitis versus samples without glomerulitis
Table S4: Glomerulitis by dominant rejection pathology
Table S5: Pathology of glomerulitis by DSA detection.
Table S6: Pathology of glomerulitis by $C4d_{ptc}$ status.
Table S7: Double-negative glomerulitis without $C4d_{ptc}$ or DSA.
Table S8: Isolated glomerulitis versus glomerulitis with inflammation.
Table S9: Detailed spatial transcriptomics results
Table S10. Determinants of graft failure.

Table S1. STROBE guidelines for cross-sectional studies. Checklist.

	Item No	Recommendation
**Title and abstract**	1	(*a*) Indicate the study's design with a commonly used term in the title or the abstract p4
		(*b*) Provide in the abstract an informative and balanced summary of what was done and what was found yes p4
**Introduction**		
Background/rationale	2	Explain the scientific background and rationale for the investigation being reported p 5-6
Objectives	3	State specific objectives, including any prespecified hypotheses p5-6
**Methods**		
Study design	4	Present key elements of study design early in the paper p6
Setting	5	Describe the setting, locations, and relevant dates, including periods of recruitment, exposure, follow-up, and data collection p6
Participants	6	(*a*) Give the eligibility criteria, and the sources and methods of selection of participants p6
Variables	7	Clearly define all outcomes, exposures, predictors, potential confounders, and effect modifiers. Give diagnostic criteria, if applicable p6 and multlple
Data sources/ measurement	8*	For each variable of interest, give sources of data and details of methods of assessment (measurement). Describe comparability of assessment methods if there is more than one group. Yes, in all
Bias	9	Describe any efforts to address potential sources of bias. P7 and elsewhere
Study size	10	Explain how the study size was arrived at. N/A
Quantitative variables	11	Explain how quantitative variables were handled in the analyses. If applicable, describe which groupings were chosen and why p7
Statistical methods	12	(*a*) Describe all statistical methods, including those used to control for confounding p7. Many multivariable analyses to handle this.
		(*b*) Describe any methods used to examine subgroups and interactions. yes
		(*c*) Explain how missing data were addressed. Omitted. Virtually no missing data
		(*d*) If applicable, describe analytical methods taking account of sampling strategy. NA
		(*e*) Describe any sensitivity analyses. In SDC multiple
**Results**		
Participants	13*	(a) Report numbers of individuals at each stage of study—eg numbers potentially eligible, examined for eligibility, confirmed eligible, included in the study, completing follow-up, and analysed. P7/8 and SDC
		(b) Give reasons for non-participation at each stage. NA
		(c) Consider use of a flow diagram. Fig 1
Descriptive data	14*	(a) Give characteristics of study participants (eg demographic, clinical, social) and information on exposures and potential confounders. P8 & SDC
		(b) Indicate number of participants with missing data for each variable of interest. NA. No missing data of note.

Outcome data	15*	Report numbers of outcome events or summary measures yes
Main results	16	(*a*) Give unadjusted estimates and, if applicable, confounder-adjusted estimates and their precision (eg, 95% confidence interval). Make clear which confounders were adjusted for and why they were included. SDC
		(*b*) Report category boundaries when continuous variables were categorized. Yes
		(*c*) If relevant, consider translating estimates of relative risk into absolute risk for a meaningful time period. NA
Other analyses	17	Report other analyses done—eg analyses of subgroups and interactions, and sensitivity analyses. Methods and SDC
**Discussion**		
Key results	18	Summarise key results with reference to study objectives. P15-16
Limitations	19	Discuss limitations of the study, taking into account sources of potential bias or imprecision. Discuss both direction and magnitude of any potential bias. P18
Interpretation	20	Give a cautious overall interpretation of results considering objectives, limitations, multiplicity of analyses, results from similar studies, and other relevant evidence. P16-18
Generalisability	21	Discuss the generalisability (external validity) of the study results. P17
**Other information**		
Funding	22	Give the source of funding and the role of the funders for the present study and, if applicable, for the original study on which the present article is based. P7. No funding

Table S2. Transcriptomic methods. Pathology of glomerulitis.

Samples
Human kidney biopsy samples were collected with consent of the patients in approved studies by the Western Sydney Local Health District Human Research Ethics Committee (2019/ETH02085 and 2025/ETH00055). During routine clinical care when transplant patients were scheduled to undergo percutaneous kidney biopsy, small segment of the biopsy core was donated to the study and stored in Dulbecco's Modified Eagle Medium (DMEM) until transferred to the PC2 facility. One sample was a wedge biopsy of an explanted kidney and transported in DMEM. The kidney biopsies were then fixed in 10% neutral buffered formalin for at last 4-hours, then transferred to 70% ethanol and underwent routine paraffin embedding.

Spatial transcriptomics data
Paraffin blocks were kept at 4°C for storage until use. Kidney samples were processed as per manufacturer's instructions for Visium HD Spatial Gene Expression User Guide (CG000685, 10x Genomics). Sectioned kidney samples (5um thickness) were stained with Haematoxylin & Eosin, imaged using the Olympus VS200 slide scanner with a 20x objective and aligned onto the VisiumHD 6.5 x 6.5mm capture areas using the Visium CytAssist. Probe-based library preparation was subsequently performed and libraries were pooled for massively parallel sequencing on the Illumina Novaseq X 10B flow cell with the number of reads per sample adjusted based on the percentage of capture area covered by the tissue. A maximum of 275 million reads is typically generated per sample for a full capture area.

Bioinformatics pipeline
Loupe Browser (V8, 10X genomics) was used to manually align bright field images with capture areas and fiducials. Spaceranger (V3.1.2, 10X genomics) was use to align and map raw reads to the spaceranger reference refdata-gex-GRCh38-2020-A and V2 human probset according to the manufacturer's instructions

Non-sclerosed glomeruli were manually selected using Loupe Browser (ver 9.0, 10x Genomics). Analysis was performed using R-studio with *Seurat* (https://www.nature.com/articles/s41587-023-01767-y) for sample loading, merge, filtering (spots > 5 features and > 5 counts, with < 50% mitochondrial DNA), normalization and data scaling for 16um bins. Dimension reduction with PCA and UMAP was performed using variable genes identified by *FindVariableFeatures*, which calculates feature variance by polynomial regression. *FindMarkers*, was used to identify differentially expressed genes (DEG) based on non-parametric Wilcoxin rank sum testing and results were filtered to keep DEG with log2-fold change > 0.5 and Benjamin Hocberg adjusted p-value < 0.1. Gene set enrichment analysis was performed on these DEG using *clusterProfiler* (doi:10.1089/omi.2011.0118.) adjusted p-value < 0.1 and results were filtered to include only immune related pathways, similar to previously described (doi: 10.1016/j.kint.2023.05.008)

Table S3. Pathology of glomerulitis. Comparison of biopsy samples with glomerulitis against samples without glomerulitis. Mean±SD, (n, %).

**Category**	**Glomerulitis**	**None**	**P value**
Biopsies (n)	271	4029	
Time (months)	31.2±53.3	18.6±39.7	<0.001
Indication biopsy (n, %)	143 (52.8)	1190 (27.3)	<0.001
Banff g score	1.3±0.5	0.0±0.0	<0.001
Banff ptc score	0.9±0.9	0.1±0.4	<0.001
MVI score (of 6)	2.0±1.1	0.1±0.4	<0.001
MVI≥2 (n, %)	142 (52.4)	81 (2.0)	<0.001
Banff i score	0.77±1.0	0.2±0.6	<0.001
Banff i-IFTA (ci0=excl.)	1.4±0.9	1.0±0.9	<0.001
Banff ti score	1.2±1.1	0.4±0.7	<0.001
Banff t-IFTA (ct0=excl.)	0.8±0.8	0.6±0.7	<0.001
Banff t score	1.0±0.8	0.4±0.6	<0.001
Banff v score	0.2±0.4	0.03±0.2	<0.001
Banff cg score	0.5±0.8	0.05±0.3	<0.001
Banff mm score	0.5±0.7	0.1±0.4	<0.001
Banff ci score	1.2±1.1	0.8±0.9	<0.001
Banff ct score	1.3±1.0	0.9±0.9	<0.001
Banff cv score	0.7±0.8	0.6±0.8	0.057
Banff ah score	0.8±1.0	0.5±0.8	<0.001
$C4d_{ptc}$ score	0.5±0.9	0.1±0.4	<0.001
$C4d_{glom}$ score	0.7±1.0	0.2±0.5	<0.001
$C4d_{art}$ score	0.3±0.6	0.1±0.3	<0.001
**Capillary endothelial ultrastructure**			
Biopsies with EM (n, %)	143 (52.8)	2079 (51.6)	
Total glomerular capillary endothelial abnormal (n, %)	98 (68.5)	783 (37.9)	<0.001
PTC-ML (maximal layers)	3.8±2.4	2.5±1.2	<0.001
**Serology results**			
Biopsies with DSA (n)	254	3352	
DSA found (MFI≥500)	129 (50.8)	1214 (36.2)	<0.001
Mean MFI (nil DSA=0)	3936±7282	1216±3350	
**Banff schema aligned diagnosis**			
Active Banff 2019 AMR	121 (44.6)	79 (2.0)	<0.001
Banff 2022 AMR (+probable)	162 (59.8)	109 (2.7)	<0.001
Banff 2022 TCMR (total)	132 (48.7)	530 (13.2)	<0.001
Borderline TCMR	49	276	
Banff 1a/b	48	170	
Banff IIa/b (v≥1)	36	70	
Banff CA-TCMR	22	14	

Table S4. Histopathology of glomerulitis by dominant pathophysiology of rejection. Etiological diagnosis. Mean±SD, (n, %).

**Category**	**TCMR**	**AMR**	**Mixed**
Biopsies (n)	68	81	112
Banff g score	1.1±0.4	1.3±0.6	1.3±0.6
Banff ptc score	0.2±0.5	0.6±0.9	1.1±1.0
MVI score (of 6)	1.3±0.6	2.0±1.1	2.5±1.1
Banff i score	0.5±0.8	0.2±0.6	1.4±0.9
Banff ti score	0.7±0.9	0.7±0.9	1.9±0.9
Banff i-IFTA (ci0=excl.)	1.1±0.8	1.1±0.9	1.9±0.9
Banff t-IFTA (ct0=excl.)	0.7±0.7	0.8±0.7	1.5±0.8
Banff t score	0.7±0.7	0.6±0.5	1.5±0.9
Banff v score	0.2±0.4	0.1±0.5	0.2±0.4
Banff cg score	0.0±0.0	0.8±1.0	0.5±0.9
Banff mm score	0.1±0.3	0.7±0.8	0.5±0.7
Banff ci score	0.7±0.9	1.1±1.0	1.6±1.0
Banff ct score	0.9±0.8	1.2±0.9	1.6±1.1
Banff cv score	0.5±0.8	0.8±0.9	0.7±0.9
Banff ah score	0.4±0.8	1.2±1.1	0.8±1.0
$C4d_{ptc}$ score	0.0±0.0	0.5±0.8	0.8±1.0
$C4d_{glom}$ score	0.1±0.3	1.0±1.1	0.9±1.0
$C4d_{art}$ score	0.2±0.5	0.2±0.5	0.5±0.7
Any $C4d_{ptc}$ (n, %)	0 (0)	29 (35.8)	54 (48.2)
Any $C4d_{glom}$ (n, %)	8 (11.8)	47 (58.0)	60 (53.6)
**Banff schema aligned diagnosis**			
Banff 2019 AMR (n, %)	0 (0)	58 (71.6)	98 (87.5)
Banff 2022 AMR (n, %)	10 (14.7)	60 (74.1)	91 (81.3)
**Serology results**			
DSA results available	63 (92.6)	79 (97.5)	103 (92.0)
No DSA found (n, % tested)	53 (84.1)	28 (35.4)	35 (34.0)
DSA found (MFI≥500)	10 (15.9)	51 (64.6)	68 (66.0)
Class I alone	5	11	18
Class II alone	4	30	33
Classes I & II	1	10	17
Mean MFI (nil DSA=0)	118±287	5153±7963	5770±8310

Table S5. Pathology of glomerulitis by DSA detection. Comparison of microvascular inflammation (glomerulitis) by detection of DSA (MFI≥500). Mean±SD, (n, %). Banff 2022 AMR includes probable. Datafile: G4a col 113

Category	Glomerulitis		
	**DSA+**	**DSA−**	**P value**
**Histopathology**			
Biopsies (n)	130	125	
Time (months)	43.2±60.7	21.0±45.1	<0.001
Indication (n, %)	79 (61.2)	60 (48.0)	0.034
Banff g score	1.3±0.6	1.2±0.5	0.059
Banff ptc score	1.0±1.0	0.4±0.8	<0.001
MVI score (of 6)	2.3±1.2	1.7±1.0	<0.001
Banff i score	0.8±1.0	0.7±1.0	0.242
Banff t score	1.1±0.8	0.9±0.8	0.013
Banff ti score	1.3±1.0	1.0±1.0	0.017
Banff i-IFTA (ci0=excl.)	1.6±1.0	1.3±0.9	0.093
Banff t-IFTA (ct0=excl.)	1.1±1.0	1.0±0.8	0.414
Banff v score	0.2±0.5	02±0.4	0.601
Banff cg score	0.7±1.1	0.2±0.5	<0.001
Banff mm score	0.6±0.8	0.3±0.6	0.002
Banff ci score	1.3±1.0	1.0±1.0	0.020
Banff ct score	1.4±0.9	1.1±1.0	0.033
Banff cv score	0.7±0.9	0.6±0.8	0.304
Banff ah score	0.9±1.1	0.7±1.0	0.371
$C4d_{ptc}$ score	0.7±1.0	0.2±0.5	<0.001
$C4d_{glom}$ score	1.0±1.0	0.5±0.8	<0.001
$C4d_{art}$ score	0.4±0.7	0.3±0.5	0.370
Any $C4d_{ptc}$ (n, %)	51 (39.2)	25 (20.0)	<0.001
Any $C4d_{glom}$ (n, %)	69 (53.1)	42 (33.6)	0.002
**Serology results**			
DSA MFI (mean)	7749±8662	0±0	<0.001
EM available (n, %)	79 (60.8)	61 (48.8)	
Glomerular capillary endothelial abnormal (n, %)	65 (82.3)	31 (50.8)	<0.001
PTC-ML (maximal layers)	4.6±2.8	2.8±1.4	<0.001
**Banff schema aligned diagnosis**			
Banff 2019 AMR (n, %)	104 (80.0)	43 (34.4)	<0.001
Banff 2022 AMR (n, %)	130 (100.0)	25 (20.0)	<0.001
Banff 2022 TCMR (n, %)	71 (54.6)	54 (43.2)	0.069
**Root cause analysis of dominant diagnosis**			
Classified biopsies (n)	130	122	
Ischemia PMN (n, %)	1 (0.8)	6 (4.9)	0.046
Pure TCMR (n, %)	10 (7.7)	53 (43.3)	<0.001
Pure AMR (n, %)	51 (39.2)	28 (23.0)	0.005
Mixed AMR/TCMR (n, %)	68 (52.3)	35 (28.7)	0.034
Any attributed AMR (n, %)	119 (91.5)	63 (51.6)	<0.001

Table S6. Histopathology & DSA of glomerulitis by $C4d_{ptc}$.

Category	Glomerulitis $C4d_{ptc}$−	Glomerulitis $C4d_{ptc}$+	P value
Biopsies (n)	188	83	
Time (months)	27.0±52.8	40.7±53.8	0.002
Indication biopsy (n, %)	93 (49.5)	50 (60.2)	0.102
$C4d_{ptc}$ score	0.0±0.0	1.6±0.8	<0.001
$C4d_{glom}$ score	0.5±0.9	1.1±1.0	<0.001
$C4d_{art}$ score	0.3±0.5	0.4±0.7	0.060
Any $C4d_{glom}$	63 (33.5)	53 (63.9)	<0.001
Banff g score	1.2±0.5	1.4±0.6	0.066
Banff ptc score	0.4±0.7	1.3±1.0	<0.001
MVI score (of 6)	1.7±0.9	2.6±1.3	<0.001
Banff i score	0.6±0.9	1.0±1.0	0.002
Banff ti score	1.0±1.0	1.5±1.1	<0.001
Banff i-IFTA (ci0=excl.)	1.3±0.9	1.7±1.0	0.007
Banff t-IFTA (ct0=excl.)	0.9±0.8	1.3±0.9	0.009
Banff t score	0.9±0.8	1.3±0.8	<0.001
Banff v score	0.1±0.4	0.2±0.6	0.095
Banff ci score	1.0±1.0	1.5±1.0	<0.001
Banff cg score	0.4±0.8	0.6±0.9	0.061
**Serology results**			
**DSA results:** available (n, %)	179 (95.2)	79 (91.6)	
DSA detected (MFI>500)	79 (44.1)	51 (64.6)	<0.001
Mean MFI (nil DSA=0)	2455±5583	7555±9368	<0.001
**EM samples:** available (n, %)	96 (51.1)	47 (56.6)	
Abnormal glomerular capillary endothelium (n, %)	57 (59.4)	41 (49.4)	<0.001
PTC-ML (maximal layers)	3.4±1.9	4.8±2.9	<0.001
**Banff schema aligned diagnosis**			
Banff 2019 AMR (n, %)	73 (38.8)	83 (100.0)	<0.001
Banff 2022 AMR (n, %)	79 (42.0)	83 (100.0)	<0.001
Banff 2022 TCMR (n, %)	75 (39.9)	57 (68.7)	<0.001
**Root cause analysis of dominant diagnosis**			
Classified biopsies (n)	185	83	
Ischemia PMN (n, %)	7 (3.8)	0 (0.0)	<0.001
Pure TCMR (n, %)	68 (36.8)	0 (0.0)	<0.001
Pure AMR (n, %)	52 (28.1)	29 (34.9)	0.261
Mixed AMR/TCMR (n, %)	58 (31.3)	83 (100.0)	<0.001
Any attributed AMR (n, %)	110 (59.4)	83 (100.0)	<0.001

Table S7. Double-negative glomerulitis without $C4d_{ptc}$ or DSA versus AMR glomerulitis. Clinical and etiological data and transplant histology of DSA negative and C4d "double negative" glomerulitis compared with $C4d_{ptc}$+ glomerulitis OR DSA_c+ glomerulitis as Banff 2022 AMR (including probable AMR). Datafile: G4aa col 113==1/0 DSA any +/- col 79 c4d versus col 165==1 AMR (c4dp+ and/or DSA+) as AMR 22 glomerulitis.
* These cases are Banff 2019 negative AMR as $C4d_{ptc}$− and DSA+ but without MVI≥2 and fails to meet all 3x AMR criteria. Key: Mean±SD, n (%).

**Category**	DSA− $C4d_{ptc}$−	DSA+ or $C4d_{ptc}$+	**P value**
LM biopsies (n)	100	155	
**Immunosuppression and preceding causes**			
Sensitized (n, %)	45 (45.0)	50 (32.3)	0.040
Early DGF ischemia (n, %)	20 (20.0)	6 (3.9)	<0.001
Iatrogenic low dose meds	26 (26.0)	53 (34.2)	0.184
Non-adherence to meds	9 (9.0)	46 (29.7)	<0.001
**Banff schema aligned diagnosis (any rejection present)**			
Banff 2022 TCMR (n, %)	36 (36.0)	89 (57.4)	<0.001
Banff 2019 AMR (n, %)	0 (0)	114 (73.5)*	<0.001
Banff 2022 AMR (including probable) (n, %)	0 (0)	100 (100.0)	<0.001

Root cause etiological analysis of dominant diagnosis COL 156
Including CD3 and CD68 glomerular immunophenotyping
Excluding n=3 unclassifiable, col 156 instead of 157.

**Root cause analysis of dominant diagnosis**			
Classified biopsies (n)	97	155	
Ischemia PMN (n, %)	6 (6.2)	1 (0.6)	<0.001
Pure TCMR (n, %)	53 (54.6)	10 (6.5)	<0.001
Pure AMR (n, %)	20 (20.6)	59 (38.1)	0.002
Mixed AMR/TCMR (n, %)	18 (18.6)	85 (54.8)	<0.001
Any attributed AMR (n, %)	38 (39.2)	144 (92.9)	<0.001
**Serology results**			
DSA results available (n, %)	100 (100)	155 (100)	
DSA detected (MFI>500)	0 (0)	130 (83.9)	<0.001
Mean MFI (nil DSA=0)	0±0	6540±8420	<0.001

Table S7 continued. Histopathology of double-negative glomerulitis versus Banff 2022 AMR glomerulitis including probable. Key: Mean±SD, n (%).

**Category**	DSA− $C4d_{ptc}$−	DSA+ or $C4d_{ptc}$+	**P value**
**LM samples** (n)	100	155	
Time (months)	17.4±40.8	41.7±60.0	<0.001
Indication biopsy (n, %)	45 (45.0)	94 (60.1)	0.014
Banff g score	1.2±0.5	1.3±0.6	0.034
Banff ptc score	0.3±0.7	0.9±1.0	<0.001
MVI score (of 6)	1.5±0.9	2.3±1.2	<0.001
Banff i score	0.6±0.9	0.9±0.9	0.018
Banff ti score	0.8±0.9	1.4±1.1	<0.001
Banff i-IFTA (ci0=excl.)	1.2±0.8	1.6±0.9	0.007
Any Banff i-IFTA (ci0=inc.)	41 (41.0)	99 (63.9)	<0.001
Banff t-IFTA (ct0=excl.)	0.9±1.2	1.2±0.9	0.018
Banff t score	0.8±0.8	1.2±0.8	<0.001
Banff v score	0.1±0.4	0.2±0.5	0.458
Banff ci score	0.9±1.0	1.3±1.0	<0.001
Banff ct score	1.0±0.9	1.4±1.0	0.003
Banff cg score	0.2±0.5	0.6±1.0	<0.001
Banff mm score	0.3±0.6	0.6±0.8	0.005
Banff cv score	0.6±0.8	0.7±0.7	0.197
$C4d_{ptc}$ score	0.0±0.0	0.78±1.0	<0.001
$C4d_{glom}$ score	0.5±0.8	1.0±1.0	<0.001
$C4d_{art}$ score	0.3±0.5	0.4±0.7	0.099
Any $C4d_{ptc}$ (n, %)	0 (0)	76 (49.0)	<0.001
Any $C4d_{glom}$ (n, %)	29 (29.0)	82 (52.9)	<0.001
**EM samples** (n, available, %)	47 (47.0)	93 (60.0)	
Abnormal glomerular capillary endothelium (n, %)	20 (42.5)	76 (81.7)	<0.001
Normal endothelium	27	17	
Minor abnormal (cg0e)	7	17	
Banff cg1a or worse	13	59	<0.001
Podocyte effacement	46 assess	89 assessable	
None (<10%)	9	14	
Mild (10%-25%)	30	44	
Moderate (26%-50%)	6	22	
Severe (>50%)	1	9	0.019
Podocyte fusion score	1.0±0.6	1.3±0.9	0.031
PTC-ML (maximal layers)	2.7±1.5	4.3±2.6	<0.001

Table S8. Isolated glomerulitis versus glomerulitis with inflammation. Presence or absence of histological inflammation (Banff ti, i, ptc, v, and cg scores all zero. Mean±SD, (n, %).

**Glomerulitis category**	**Isolated**	**Inflamed**	**P value**
Biopsies (n)	55	216	
Post-transplant time (months)	6.6±12.7	37.4±57.8	<0.001
Protocol diagnosis (n, %)	39 (70.9)	89 (41.2)	<0.001
Banff g score	1.2±0.4	1.3±0.6	0.061
Banff ptc score	0.0±0.0	0.9±1.0	<0.001
MVI score (of 6)	1.2±0.4	2.2±1.2	<0.001
Banff i score	0.0±0.0	1.0±1.0	<0.001
Banff ti score	0.0±0.0	1.4±1.0	<0.001
Banff i-IFTA (ci0=excl.)	0.7±0.8	1.6±0.9	<0.001
Banff t-IFTA (ct0=excl.)	0.7±0.8	1.6±0.9	<0.001
Banff t score	0.4±0.5	1.1±0.8	<0.001
Banff v score	0.0±0.0	0.2±0.5	<0.001
Banff cg score	0.0±0.0	0.6±0.9	<0.001
Banff mm score	0.2±0.4	0.5±0.7	<0.001
Banff ci score	0.4±0.6	1.4±1.1	<0.001
Banff ct score	0.6±0.6	1.4±1.0	<0.001
Banff cv score	0.3±0.6	0.7±0.9	<0.001
Banff ah score	0.4±0.7	0.9±1.0	<0.001
$C4d_{ptc}$ score	0.2±0.4	0.6±0.9	<0.001
$C4d_{glom}$ score	0.3±0.6	0.8±1.0	<0.001
$C4d_{art}$ score	0.2±0.4	0.3±0.6	0.045
Any $C4d_{ptc}$ (n, %)	8 (14.5)	75 (34.7)	0.004
Any $C4d_{glom}$ (n, %)	11 (20.0)	105 (48.6)	<0.001
**Serology results**			
DSA results available	48	207	0.016
DSA+ found (MFI≥500)	20 (41.7)	110 (53.1)	0.153
Mean MFI (nil DSA=0)	810±1572	4709±7886	
EM: Samples available (n)	25	118	
Glom. endothelium abnormal	9 (16.4)	89 (75.4)	<0.001
Average PTC-ML (maximal)	2.2±1.1	4.2±2.5	<0.001
**Banff schema aligned diagnosis**			
Banff 2019 AMR (n, %)	9 (16.4)	147 (68.1)	<0.001
Banff 2022 AMR (n, %)	23 (41.8)	139 (64.4)	0.002
Banff 2022 TCMR (n, %)	1 (1.8)	131 (60.6)	<0.001
**Root cause analysis:** (n)	54	214	
Ischemia PMN (n, %)	6 (11.1)	1 (0.5)	<0.001
Pure TCMR (n, %)	28 (51.9)	40 (18.7)	<0.001
Pure AMR (n, %)	19 (35.2)	62 (29.0)	0.375
Mixed AMR/TCMR (n, %)	1 (1.9)	111 (51.9)	<0.001

Table S9. Detailed spatial transcriptomics results. Genes that belong to the Banff Human Organ Transplant (BHOT) consensus gene panel are annotated in red and bolded.

Description of the biopsy samples and number of glomeruli in the spatial analysis

Samples	Gloms	Sample information
No rejection	7	Two biopsies, both with i0 t0 g0 ptc0 v0 c4d0 and no DSA detected (one biopsy at 1-month post and one biopsy 12-months post kidney transplantation)
Isolated glomerulitis	5	Two 1-month post-kidney transplantation protocol biopsies, both with g1 ptc0 i0 t0 c40 and no DSA
Mixed Rejection	12	Transplant nephrectomy sample for an acutely inflamed graft. The patient was already on regular hemodialysis with a failed kidney transplant from chronic mixed rejection, but required 1500mg methylprednisolone (3 divided doses) for acute graft pain following ipilimumab for native kidney renal cell carcinoma. Banff scores ti3 g1 ptc2 t2 v3 ci3 ct3 cv3 cg2 i-IFTA3 c4d1. DSA status N/A.

Supplementary table
Number of and top 10 differentially expressed genes (DEG) for each comparison group which had adjusted P-value < 0.1 and log_2-FC > 0.5

Comparison	DEG	Top 10 differentially expressed gene (DEG) with $Log_2FC > 0.5$ adj p-value < 0.1
Isolated glomerulitis vs no rejection	1521	ALDOB, THY1, GPC3, UTY, ARF5, EIF1AY, **MIF**, CD164, CDH16, EPCAM
Isolated glomerulitis vs mixed rejection	1352	ADLOB, SCL12A, SOST, IL13RA2, MT-ATP6, HBB, MT-ND4, MT-CO3
Mixed rejection vs no rejection	343	**C1QB**, C1QC, **SERPINE1**, CD163, **C1QA**, DDIT4, ERFF1, TNFRSF12A, CEBPD, **MT2A**

DEG with $Log_2FC > 0.5$, Adjusted P < 0.1

Cluster	DEG	Top 10 differentially expressed gene (DEG) with $Log_2FC > 0.5$ adj p-value < 0.1
0	275	APLN, ESM1, ADORA1, TSPAN2, NT5E, **NPHS2**, TYRO3, ALS2CL, RETREG1, NTNG1
1	172	EXOC3L2, PTPRB, ADCY4, **TEK**, GJA5, LDB2, **SOST**, TBX3, EMID1, EMCN
2	396	**COL1A1**, C1QA, LYZ, **CD163**, **C1QB**, TYROBP, C1QC, MARCKS, **COL3A1**, SAT1
3	158	IGSF9B, C3, SLP1, MMP7, CLDN1, MYRF, CDH6, LAD1, RAPGAP, BHLHE41
4	1734	APLNR, ADCY1, IL13RA, FCN3, NSG1, CPXM1, FLI1, JPH4, RAMP2, JPH4, CHODL
5	75	ALDOB, CALB1, CDH16, **SLC12A**, MT-ATP6, **SLC4A**, HBB, MT-CO3, MT-ND4, MT-CO2

Table S10. Selected univariable and multivariable determinants of graft failure. Univariable Cox regression predictors of death-censored allograft failure (n=204 kidneys with glomerulitis from the time of index biopsy, 1st occurrence of glomerulitis).

	HR	95%CI	P value
**Univariable clinical risk factors**			
Biopsy time (months)	1.009	1.006-1.012	<0.001
Late presentation	1.688	2.585-11.321	<0.001
Indication biopsy	6.556	2.781-15.457	<0.001
Recipient age (years)	0.952	0.930-0.974	<0.001
Recipient male	0.631	0.355-1.120	0.166
Pre-sensitization	0.429	0.207-0.890	0.023
Living donor	1.760	0.979-3.164	0.059
HLA mismatch (n/6)	1.035	0.862-1.244	0.712
Delayed function	0.949	0.472-1.908	0.949
Early cellular rejection	1.862	1.017-3.407	<0.001
Early antibody rejection	2.687	1.252-5.765	0.011
Prior IV corticosteroids	2.195	1.222-3.943	0.009
Prior ATG (any)	3.236	1.770-5.914	<0.001

	HR	95%CI	P value
**Univariable pathological and antibody risk factors**			
Banff g score	1.081	0.640-1.825	0.771
Banff ptc score	1.689	1.290-2.212	<0.001
MVI score (of 6)	1.432	1.155-1.775	<0.001
Banff ti score	1.448	1.120-1.874	0.005
Banff i score	1.181	0.899-1.551	0.232
Banff t score	1.343	0.968-1.862	0.077
Banff i-IFTA (n=141)	1.367	0.981-1.904	0.064
Banff t-IFTA	1.185	0.838-1.675	0.338
Banff cg score	2.020	1.563-2.611	<0.001
Banff mm score	2.375	1.688-3.342	<0.001
Banff ci score	2.507	1.859-3.381	<0.001
Banff ct score	2.713	1.982-3.714	<0.001
Banff cv score	2.086	1.506-2.888	<0.001
$C4d_{ptc}$ score	1.906	1.459-2.491	<0.001
$C4d_{ptc}$ (any)	2.214	1.240-3.954	0.007
$C4d_{glom}$ score	1.900	1.476-2.447	<0.001
$C4d_{glom}$ (any)	2.618	1.146-4.685	<0.001
$C4d_{art}$ score	1.228	0.721-2.091	0.449
$C4d_{art}$ (any)	1.276	0.659-2.468	0.470
DSA (≥500 MFI, any)	2.809	1.467-5.379	0.002
DSA (ln[MFI+1])	1.206	1.107-1.314	<0.001

Table S10 continued. Selected univariable and multivariable determinants of graft failure. Univariable Cox regression predictors of death-censored allograft failure (n=204 kidneys with glomerulitis from the time of index biopsy, 1st occurrence of glomerulitis). Histopathological model 1 restricted to pathology and donor specific antibody (DSA). Clinical and pathological mixed model 2 includes renal function and proteinuria.

Multivariable risk factors: Model 1
Histopathological restricted model including DSA.

	HR	95%CI	P value
DSA ln(MFI+1)	1.105	1.010-1.209	0.030
$C4d_{ptc}$ score	1.441	1.078-1.927	0.014
Banff ci score	2.364	1.691-3.305	<0.001
Banff cg score	1.446	1.064-1.965	0.018

Multivariable risk factors: Model 2:
Parsimonious clinical and pathological mixed model.

	HR	95%CI	P value
S. creatinine (umol/l)	1.002	1.001-1.002	<0.001
Urinary albumin/creatinine (mg/mmol)	1.004	1.002-1.006	<0.001
DSA ln(MFI+1)	1.129	1.025-1.243	0.014
Banff ci score	2.264	1.793-3.892	<0.001
